# Zika Virus Emergence in Mosquitoes in Southeastern Senegal, 2011

**DOI:** 10.1371/journal.pone.0109442

**Published:** 2014-10-13

**Authors:** Diawo Diallo, Amadou A. Sall, Cheikh T. Diagne, Oumar Faye, Ousmane Faye, Yamar Ba, Kathryn A. Hanley, Michaela Buenemann, Scott C. Weaver, Mawlouth Diallo

**Affiliations:** 1 Unité d’Entomologie Médicale, Institut Pasteur de Dakar, Dakar, Sénégal; 2 Unité des Arbovirus et Virus des Fièvres Hémorragiques, Institut Pasteur de Dakar, Dakar, Sénégal; 3 Department of Biology, New Mexico State University, Las Cruces, New Mexico, United States of America; 4 Department of Geography, New Mexico State University, Las Cruces, New Mexico, United States of America; 5 Institute for Human Infections and Immunity, Center for Tropical Diseases, and Department of Pathology, University of Texas Medical Branch, Galveston, Texas, United States of America; The Pirbright Institute, United Kingdom

## Abstract

**Background:**

Zika virus (ZIKV; genus *Flavivirus*, family *Flaviviridae*) is maintained in a zoonotic cycle between arboreal *Aedes* spp. mosquitoes and nonhuman primates in African and Asian forests. Spillover into humans has been documented in both regions and the virus is currently responsible for a large outbreak in French Polynesia. ZIKV amplifications are frequent in southeastern Senegal but little is known about their seasonal and spatial dynamics. The aim of this paper is to describe the spatio-temporal patterns of the 2011 ZIKV amplification in southeastern Senegal.

**Methodology/Findings:**

Mosquitoes were collected monthly from April to December 2011 except during July. Each evening from 18∶00 to 21∶00 hrs landing collections were performed by teams of 3 persons working simultaneously in forest (canopy and ground), savannah, agriculture, village (indoor and outdoor) and barren land cover sites. Mosquitoes were tested for virus infection by virus isolation and RT-PCR. ZIKV was detected in 31 of the 1,700 mosquito pools (11,247 mosquitoes) tested: *Ae. furcifer* (5), *Ae. luteocephalus* (5), *Ae. africanus* (5), *Ae. vittatus* (3), *Ae. taylori*, *Ae. dalzieli*, *Ae. hirsutus* and *Ae. metallicus* (2 each) and *Ae. aegypti*, *Ae. unilinaetus*, *Ma. uniformis*, *Cx. perfuscus* and *An. coustani* (1 pool each) collected in June (3), September (10), October (11), November (6) and December (1). ZIKV was detected from mosquitoes collected in all land cover classes except indoor locations within villages. The virus was detected in only one of the ten villages investigated.

**Conclusions/Significance:**

This ZIKV amplification was widespread in the Kédougou area, involved several mosquito species as probable vectors, and encompassed all investigated land cover classes except indoor locations within villages. *Aedes furcifer* males *and Aedes vittatus* were found infected within a village, thus these species are probably involved in the transmission of Zika virus to humans in this environment.

## Introduction

Zika virus (ZIKV; genus *Flavivirus*, family *Flaviviridae*) is transmitted in a zoonotic cycle between arboreal *Aedes* spp. mosquitoes and nonhuman primates in African and Asian forests [1], [2]. This virus is closely related to the other *Flaviviridae* of public health importance including dengue, yellow fever, West Nile and Japanese encephalitis viruses [3], [4]. ZIKV was first isolated in Uganda from a febrile sentinel rhesus monkey and from the mosquito, *Aedes africanus,* in 1947 and 1948, respectively [5]. These initial identifications were followed by detection of ZIKV infection of humans, mosquitoes and animals in Africa and Asia by virus isolation and serological studies [6]–[11].

Although ZIKV predominantly circulates in sylvatic habitats, it has been isolated in urban settings from humans and *Ae. aegypti* in Africa and Asia [6], [10], [12]. A serological study in Nigeria showed that 40% of an urban population had neutralizing antibodies to ZIKV [6]. Moreover *Ae. aegypti* from Nigeria, Senegal and Singapore have been shown experimentally to be competent vectors of ZIKV [13]–[16]. Human infections were first described in 1964 by a medical entomologist who was infected by ZIKV during fieldwork in Uganda, and are mainly characterized by mild headaches, maculopapular rash, fever, malaise, conjunctivitis, and arthralgia [4], [17]. These observations strongly suggested the occurrence of urban ZIKV transmission, but the first epidemic was not documented until 2007 on Yap Island within the Federated State of Micronesia [4]. ZIKV infected about 73% of the Yap population in 4 months during this outbreak, which occurred outside its previously documented geographic range. Currently ZIKV is causing a large outbreak in French Polynesia [18].

In Senegal, ZIKV was first isolated from *Ae. luteocephalus* collected in 1968 in the Saboya Forest in the western part of the country, 187 km from the capital city Dakar [19], [20]. One year later, the virus was isolated from mosquitoes (*Ae. luteocephalus*, *Ae. furcifer-taylori* and *An. gambiae s.l*.) and a human in Bandia, located 65 km from Dakar. In southeastern Senegal, more than 400 ZIKV strains have been isolated from mosquitoes, mainly from *Ae. africanus, Ae. luteocephalus, Ae. furcifer,* and *Ae. taylori*. Infection of seven humans and two nonhuman primates (*Chlorocebus sabaeus*, *Erythrocebus patas*) were detected by virus isolation. Serological studies conducted in the same region in 1988 and 1990 have shown that 10.1 and 2.8% of humans had IgM antibodies against ZIKV [2].

In 2008, a research program was initiated to investigate the mechanisms of sylvatic transmission of arboviruses in Kédougou, southeastern Senegal. The environmental factors that influence the abundance, distribution and infection of mosquito vectors that participate in the sylvatic cycles of several arboviruses were investigated beginning in June 2009. We recently reported the distribution and abundance of adult mosquitoes potentially involved in the sylvatic cycle of chikungunya virus (CHIKV), as well as rates of CHIKV infection in these mosquitoes, in the five most abundant land cover classes (forest, savannah, agriculture, barren and village) occurring in an area of 1,650 km^2^ around the town of Kédougou [21]. Potential vectors and mosquito pools containing CHIKV were found in each of the land cover classes. *Ae. furcifer* was the only species present in all land covers and accounted for more than a third of CHIKV-positive mosquito pools. This species also entered in villages to feed on humans. *Ae. furcifer* was therefore considered to be the most important bridge vector between sylvatic CHIKV amplification and human populations. This outbreak was followed by an outbreak of YFV in 2010, and an amplification of ZIKV in 2011.

Despite repeated ZIKV amplifications in the Kédougou area over the past 50 years, little is known about its seasonal and spatial dynamics. Here, we describe spatial and temporal pattern of the 2011 ZIKV amplification, representing initial steps to build a more effective and predictive risk model of ZIKV amplifications in Senegal that ultimately can be used to implement better control strategies.

## Methods

### Ethics statement

The University of Texas Medical Branch (UTMB) Institutional Animal Care and Use Committee approved the animal experiments described in this paper under protocol 02-09-068. UTMB complies with all applicable regulatory provisions of the U.S. Department of Agriculture (USDA)-Animal Welfare Act; the National Institutes of Health (NIH), Office of Laboratory Animal Welfare-Public Health Service (PHS) Policy on Humane Care and Use of Laboratory Animals; the U.S Government Principles for the Utilization and Care of Vertebrate Animals Used in Research, Teaching, and Testing developed by the Interagency Research Animal Committee (IRAC), and other federal statutes and state regulations relating to animal research. The animal care and use program at UTMB conducts reviews involving animals in accordance with the *Guide for the Care and Use of Laboratory Animals* (2011) published by the National Research Council.

Mice were housed in standard, ALAAC-approved caging within the Institut Pasteur BSL-2 vivarium, one litter and mother per cage. They were housed in a light/temperature/humidity-controlled environment: 12-h light–dark cycle, temperature 22°C and 50% humidity and had *ad libitum* access to food and water. There is no way to detect pain or distress in newborn mice.

### Study area and Mosquito Sampling

**Figure 1:**
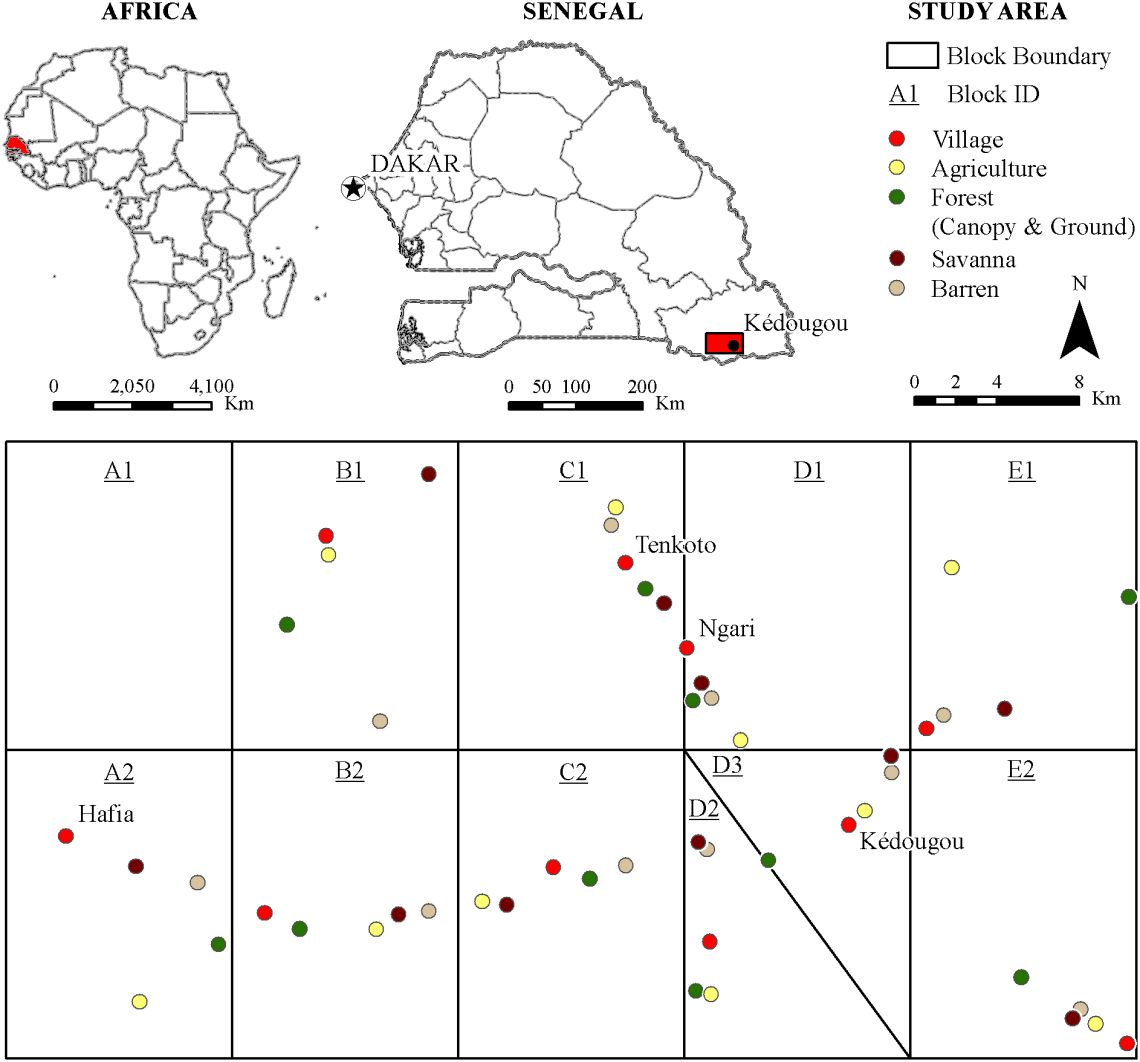
The study area. The rectangle in the upper right map corresponds to the 1,650 km^2^ divided in ten blocks (A2, B1, B2, C1, C2, D1, D2, D3, E1 and E2) below. Data were collected in each of the five land covers indicated by colored circles [agriculture, barren, village (indoor and outdoor), savannah and forest (canopy and ground)] in the ten blocks. The diagonal line separates the blocks D2 et D3 which replaced block A1, which was abandoned due to inaccessibility.

The study was conducted in the Kédougou region (Figure 1 and Table S1) of southeastern Senegal (12°33′ N, 12°11′ W); the environment, climate, and socioeconomic conditions of this region, which lies in a transition zone between the dry tropical forest and the savanna belt, has been described in detail elsewhere [21]. Deforestation for cultivation, gold mining and human habitations is progressively reducing the natural vegetation in the area. The mosquito sampling protocol was extensively described by Diallo et al. [21]. Briefly, an area of 1,650 km^2^ (30 km in N–S direction; 55 km in E–W direction) of the Kédougou region was divided into ten blocks. In each block, 5 different land cover classes (forest, barren, savannah, agriculture and village) were mapped using remote sensing. Mosquitoes were collected from one site per land cover class in each of the 10 blocks (50 sites total) monthly from April to December, except July, by human landing catch. Each evening (from 1800–2100 hrs), collections were performed by teams of 3 persons working in parallel in forest (canopy and ground), savannah, agriculture, village (indoor and outdoor) and barren sites. In the field laboratory, mosquitoes were sorted, identified and pooled by species, sex and collection site on a chill table using morphological identification keys [22]–[28]. Mosquito pools were frozen, shipped to the main laboratory in Dakar, and screened for viral infection.

The study protocol was carefully explained to the chief and inhabitants of each village investigated to obtain their informed oral consent. Informed oral consent was also obtained from the heads of each household and agricultural land cover in which collection were undertaken. The first author should be contacted for future permissions. No specific permissions were required for collection in forests, savannah and barren land covers. The field studies did not involve endangered or protected species.

### Detection of viruses in mosquito pools

Mosquito pools were homogenized in 2.5 ml of Leibovitz 15 cell culture medium containing 20% fetal bovine serum (FBS) and centrifuged for 20 min at 10,000×g at 4°C. One ml of the supernatant was inoculated into AP-61 (*Ae. pseudoscutellaris*) or Vero African green monkey kidney cells as described previously [29]. Cells were incubated at 28°C (AP-61) or 37°C (Vero), and cytopathogenic effects recorded daily. Within 10 days, slides were prepared for immunofluorescence assay (IFA) using 7 pools of immune ascitic fluids specific for most African mosquito-borne arboviruses for identification. Virus identification was completed using complement fixation and seroneutralization tests by intracerebral inoculation into newborn mice, as approved by the UTMB Institutional Animal Care and Use Committee.

For real-time RT-PCR assays, 100 µl of supernatant were used for RNA extraction with the QiaAmp Viral RNA Extraction Kit (Qiagen, Heiden, Germany) according to the manufacturer’s protocol. The RNA was amplified using a real-time RT-PCR assay and an ABI Prism 7000 SDS Real-Time apparatus (Applied Biosystems, Foster City, CA) using the Quantitect kit (Qiagen, Hilden, Germany). The 25 µl reaction volume contained 1 µl of extracted RNA, 2x QuantiTect Probe, RT-Master Mix, 10 µM of each primer and the probe. The primers and sequences probes were described by Faye et al. [30].

### Data Analysis

The pooled infection rate program (PooledInfRate, version 3.0, Center for Disease Control and Prevention, Fort Collins, CO: http://www.cdc.gov/ncidod/dvbid/westnile/software.htm) was used to calculate minimum infection rates for the species found positive for ZIKV. The entomological inoculation rate (EIR) is a measure of exposure to infectious mosquitoes. EIR was calculated as the product of the mean biting rate multiplied by the minimum infection rate. It is interpreted as the number of infective bites received by an individual over a defined time period. The entomologic inoculation rate was defined here as the number of infective mosquito bites per human per evening. The chi-square test was used to test significance of differences in rates and the Kendall rank order correlation (tau) to test the association between vector biting and infection rates (direct and with a lag time of one month) using R [31].

## Results

### Virus isolations

Thirty-one ZIKV infected pools were collected in June (3/9.7%), September (10/32.2%), October (11/35.5%), November (6/19.3%) and December (1/3.2%) of 2011 (Table 1). Overall, 31 of the 1,700 mosquito pools (comprising a total of 11,247 mosquitoes) tested were positive for ZIKV (Table 2 and Table S2). The infected pools were distributed among vector species as follows: *Ae. furcifer* (4 pools of females and 1 pool of males (the only infected male pool)/overall 16.1% of the infected pools), *Ae. luteocephalus* (5/16.1%), *Ae. africanus* (5/16.1%), *Ae. vittatus* (3/9.7%), *Ae. taylori*, *Ae. dalzieli*, *Ae. hirsutus* and *Ae. metallicus* (2/6.4% each) and finally *Ae. aegypti*, *Ae. unilinaetus*, *Ma. uniformis*, *Cx. perfuscus* and *An. coustani* (1 pool each). ZIKV was detected from mosquitoes collected in all land cover classes except in villages at indoor locations (Table 3 and Table S3), including forest canopy (10 of 31 positive pools), forest ground (12), savannah (2), barren (2), agricultural (3) and village (1 female and 1 male pool).

**Table 1:** Temporal dynamics of biting, infection and entomological inoculation rates of potential Zika virus vectors, Kédougou, 2011.

	No. Collected (No. Positive pools)					Mean biting rate					Minimum infection rate					Entomological inoculation rate				
	June	Sep	Oct	Nov	Dec	June	Sep	Oct	Nov	Dec	June	Sep	Oct	Nov	Dec	June	Sep	Oct	Nov	Dec
*Aedes aegypti*	86 (0)	14 (1)	17 (0)	6 (0)	0 (0)	0.41	0.07	0.08	0.03	0.00	0.00	71.43	0.00	0.00	0.00	0.000	0.005	0.000	0.000	0.000
*Aedes africanus*	11 (0)	168 (1)	74 (1)	82 (3)	14 (0)	0.05	0.80	0.35	0.39	0.07	0.00	5.95	13.51	36.59	0.00	0.000	0.005	0.005	0.014	0.000
*Aedes dalzieli*	39 (0)	241 (1)	1106 (0)	289 (1)	13 (0)	0.19	1.15	5.27	1.38	0.06	0.00	4.15	0.00	3.46	0.00	0.000	0.005	0.000	0.005	0.000
*Aedes furcifer*	13 (1)	802 (2)	675 (1)	97 (0)	6 (0)	0.06	3.82	3.21	0.46	0.03	76.92*	2.49	2.96	0.00	0.00	0.005	0.010	0.010	0.000	0.000
*Aedes luteocephalus*	64 (0)	387 (1)	206 (3)	34 (1)	4 (0)	0.30	1.84	0.98	0.16	0.02	0.00	2.58	14.56	29.41	0.00	0.000	0.005	0.014	0.005	0.000
*Aedes metallicus*	6 (0)	0 (0)	71 (2)	1 (0)	0 (0)	0.03	0.00	0.34	0.00	0.00	0.00	0.00	28.17	0.00	0.00	0.000	0.000	0.010	0.000	0.000
*Aedes taylori*	8 (0)	75 (0)	93 (0)	104 (1)	8 (1)	0.04	0.36	0.44	0.50	0.04	0.00	0.00	0.00	9.62	125.00	0.000	0.000	0.000	0.005	0.005
*Aedes vittatus*	675 (1)	149 (2)	145 (0)	17 (0)	5 (0)	3.21	0.71	0.69	0.08	0.02	1.48	13.42	0.00	0.00	0.00	0.005	0.010	0.000	0.000	0.000
*Aedes hirsutus*	1 (0)	2 (0)	25 (2)	4 (0)	1 (0)	0.00	0.01	0.12	0.02	0.00	0.00	0.00	80.00	0.00	0.00	0.000	0.000	0.010	0.000	0.000
*Aedes unilineatus*	5 (1)	26 (0)	33 (0)	6 (0)	1 (0)	0.02	0.12	0.16	0.03	0.00	200.00	0.00	0.00	0.00	0.00	0.005	0.000	0.000	0.000	0.000
*Anopheles coustani*	0 (0)	494 (0)	125 (1)	37 (0)	11 (0)	0.00	2.35	0.60	0.18	0.05	0.00	0.00	8.00	0.00	0.00	0.000	0.000	0.005	0.000	0.000
*Culex perfuscus*	0 (0)	7 (1)	15 (0)	0 (0)	0 (0)	0.00	0.03	0.07	0.00	0.00	0.00	142.86	0.00	0.00	0.00	0.000	0.005	0.000	0.000	0.000
*Mansonia uniformis*	0 (0)	32 (1)	98 (0)	109 (0)	37 (0)	0.00	0.15	0.47	0.52	0.18	0.00	31.25	0.00	0.00	0.00	0.000	0.005	0.000	0.000	0.000
Vectors	908 (3)	2397 (10)	2683 (10)	786 (6)	100 (1)	4.32	11.41	12.78	3.74	0.48	3.30	4.17	4.10	7.63	10.00	0.014	0.048	0.052	0.029	0.005

Mean biting rate (number of mosquito females captured per person, per evening); *Minimum infection rate (estimated number of positive mosquitoes per 1000 mosquitoes tested); for each species minimum infection rates with an asterisk are statistically significantly higher than species with an asterisk; Entomologic inoculation rate (number of infected mosquito bites per person, per evening).

**Table 2:** Mosquitoes collected and Zika virus infection of potential vectors, Kédougou, 2011.

Species	Totalcollected	Proportionof thecollection (%)	Femalescollected	Proportionof thecollection (%)	Positivefemalepools	Minimuminfection rate (‰)
*Aedes aegypti*	250	2.22	245	2.20	1	4.08
*Aedes africanus*	505	4.49	505	4.54	5	9.90*
*Aedes dalzieli*	1718	15.27	1718	15.44	2	1.16
*Aedes furcifer*	2966	26.37	2939	26.42	5**	1.36
*Aedes hirsutus*	34	0.30	34	0.30	2	58.82*
*Aedes luteocephalus*	1259	11.19	1259	11.32	5	3.97
*Aedes metallicus*	81	0.72	81	0.73	2	24.69*
*Aedes taylori*	422	3.75	395	3.55	2	5.06
*Aedes unilineatus*	38	0.34	38	0.34	1	26.31*
*Aedes vittatus*	1790	15.91	1728	15.53	3	1.74
*Anopheles coustani*	710	6.31	710	6.38	1	1.41
*Culex perfuscus*	22	0.19	22	0.20	1	45.45*
*Mansonia uniformis*	283	2.52	281	2.52	1	3.56
Others	1169	10.39	1169	10.51	0	
Total	11247		11124		30	

Minimum infection rate (estimated number of positive mosquitoes per 1000 mosquitoes tested), *Minimum infection rate with an asterisk are statistically significantly higher, **Five ZIKV isolates including 1 pool of males positive.

Others: *Ae. argenteopunctatus, Ae. centropunctatus, Ae. cumminsii, Ae. cozi, Ae. fowleri, Ae. mcintoshi,, Ae. minutus, Ae. neoafricanus, Ae. ochraceus, Ae. vexans, An. brohieri, An. funestus, An. domicola, An. flavicosta, An. freetownensis, An. gambiae* s.l., *An. hancocki, An. nili, An. pharoensis, An. rufipes, An. squamosus, An. ziemanni, Cx. Annulioris, Cx. antennatus, Cx. bitaeniorhynchus, Cx. cinerus, Cx. decens, Cx. duttoni, Cx. ethiopicus, Cx. neavei, Cx. poicilipes, Cx. quinquefasciatus, Cx. tritaeniorhynchus, Eretmapodites quinquevittatus, Ma. Africana, Fi. circumtestea.*

**Table 3:** Mosquitoes collected and Zika virus infection of potential vectors among land cover classes, Kédougou, 2011.

Species	Landcover	Femalescollected	Meanbitingrate	No.Positivepools	Minimuminfectionrate (‰)	Entomologicalinoculation rate
*Aedes aegypti*	Forest ground	75	0.50	1	13.33	0.01
*Aedes africanus*	Forest canopy	214	1.43	3	14.02	0.02
	Forest ground	133	0.89	2	15.04	0.01
*Aedes dalzieli*	Forest ground	335	2.23	1	2.99	0.01
	Agriculture	546	3.64	1	1.83	0.01
*Aedes furcifer*	Forest canopy	693	4.62	2	2.89	0.01
	Forest ground	170	1.13	2	11.76	0.01
*Aedes luteocephalus*	Forest canopy	502	3.35	3	5.98	0.02
	Forest ground	139	0.93	2	14.39	0.01
*Aedes metallicus*	Savannah	25	0.17	1	40.00	0.01
	Barren	10	0.07	1	100.00	0.01
*Aedes taylori*	Forest canopy	264	1.76	1	3.79	0.01
	Barren	6	0.04	1	166.67*	0.01
*Aedes vittatus*	Forest canopy	30	0.20	1	33.33	0.01
	Forest ground	176	1.17	1	5.68	0.01
	Village outdoor	30	0.20	1	33.33	0.01
*Aedes hirsutus*	Forest ground	5	0.03	1	200.00	0.01
	Agriculture	8	0.05	1	125.00	0.01
*Aedes unilineatus*	Forest ground	4	0.03	1	250.00	0.01
*Anopheles coustani*	Barren	91	0.61	1	10.99	0.01
*Culex perfuscus*	Agriculture	3	0.02	1	333.33	0.01
*Mansonia uniformis*	Forest ground	19	0.13	1	52.63	0.01
All vectors	Forest canopy	1761	11.74	10	5.68	0.07
	Forest ground	1125	7.50	12	10.67*	0.08
	Savannah	1259	8.39	2	0.79	0.01
	Barren	881	5.87	2	3.41	0.02
	Agriculture	1375	9.17	3	2.18	0.02
	Village outdoor	378	2.52	1	2.65	0.01
	Village indoor	95	0.63	0	0.00	0.00

Mean biting rate (number of mosquito females captured per person per evening); Minimum infection rate (estimated number of positive mosquitoes per 1000 mosquitoes tested); For each species *Minimum infection rates with an asterisk are statistically significantly higher that those without an asterisk; Entomologic inoculation rate (number of infected mosquito bites per person per evening).

**Figure 2:**
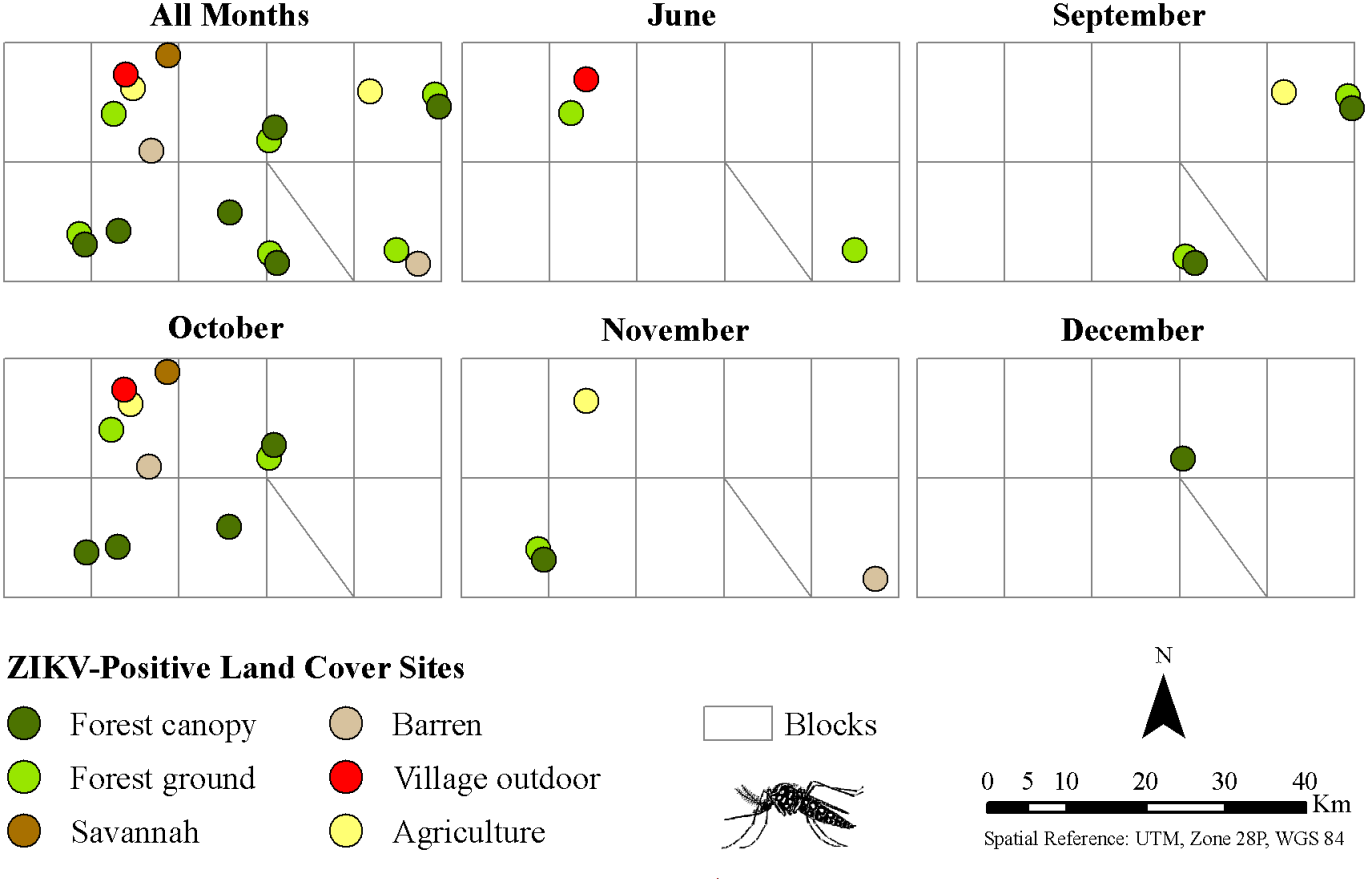
Land cover sites with positive ZIKV mosquito pools, southeastern Senegal, 2011.

To assess variation among land cover classes, each site was coded as positive (at least one ZIKV-positive pool) or negative (no ZIKV-positive pools). Based on this coding, there was a significant association between land cover classes and presence of ZIKV (χ2 = 18.7, df = 6, P = 0.005), with the forest canopy and forest ground classes significantly more likely to yield positive pools than the others (Figure 2).

### Minimum infection rates

Overall infection rates among species differed significantly (χ2 = 82.1, df = 12, P<0.0001). *Ae. furcifer*, *Ae. vittatus*, *Ae. taylori*, *Ae. luteocephalus*, *Ae. dalzieli*, *Ae. aegypti, Ma. uniformis* and *An. coustani* had the lowest (compared to *Ae. africanus, Ae. hirsutus, Ae. metallicus, Ae. unilineatus* and *Cx. perfuscus*) and statistically comparable infection rates. (χ2 = 6.4, df = 7, P = 0.5).

Infection rates of *Ae. africanus*, *Ae. furcifer*, *Ae. luteocephalus, Ae. vittatus, Ae. dalzieli* and *Ae. taylori* showed temporal and spatial variation (Tables 1 and 3). The highest infection rates were observed in June for *Ae. furcifer*, in September for *Ae. vittatus*, in November for *Ae. africanus* and *Ae. luteocephalus,* and in December for *Ae. taylori*. The temporal variation among months was statistically significant only for *Ae. furcifer* (χ2 = 27.1 df = 2, P<0.0001). Minimum infection rates of the positive land cover classes were statistically indistinguishable for all the vector species except *Ae. taylori* (χ2 = 4.8, df = 1, P = 0.03) and the combined vectors (χ2 = 16.4, df = 5, P = 0.006). The highest minimum infection rate was observed in the barren class for *Ae. taylori* and on the forest ground for the overall vectors.

There was a positive and significant correlation between biting and infection rates only for *Ae. luteocephalus* (tau = 0.7, P = 0.04) and *Ae. taylori* (tau = 0.9, P = 0.002) with a lag time of one month.

### Entomological inoculation rates

The highest mean entomological inoculation rates were those of *Ae. luteocephalus* and *Ae. africanus* in the forest canopy (Table 3). Assuming that all infected mosquitoes were capable of transmission, transmission by three vectors was likely in June, by eight in September, by six October, by four in November and by one in December (Table 1). The association of species involved in the transmission varied each month. *Ae. africanus*, *Ae. furcifer* and *Ae. luteocephalus* were involved in 3 of the 5 associations, *Ae. vittatus, Ae. dalzieli* and *Ae. taylori* in 2 associations and the other vectors (*Ae. aegypti, Ae. hirsutus, Ae. unilineatus, Ae. metallicus, Cx. perfuscus, An. coustani* and *Ma. uniformis*) in only one association. Spatially, the highest inoculation rates were observed on the forest ground in June, forest ground and forest canopy in September, forest canopy in October, and forest ground in November. Transmission was likely in December only in the forest.

Our data indicate that, between June and September to December, an individual might have received at least 10 infective bites in the forest canopy [from *Ae. africanus* (3 infective bites), *Ae. luteocephalus* (3), *Ae. furcifer* (2), *Ae. taylori* (1) and *Ae. vittatus* (1)]. There would be 12 infective bites in the forest ground, 3 on barren land and agricultural settings, and one in savannah land covers. These infective bites would be predicted to come from *Ae. africanus, Ae. luteocephalus* (3 each), *Ae. furcifer* (2) and *Ae. aegypti, Ae. dalzieli, Ae. hirsutus, Ae. unilineatus, Ae. vittatus and Ma. uniformis* (1 each) in the forest ground, from *Ae. metallicus* in the savannah, from *Ae. dalzieli*, *Ae. hirsutus* and *Cx. perfuscus* in agricultural settings, and from *Ae. metallicus*, *Ae. taylori* and *An. coustani* in the barren areas. *Ae. vittatus* would be involved in transmission within the village class outdoors where a human in this land cover might have received during the transmission season at least one infective bite from this species.

## Discussion

Following the amplification of CHIKV and YFV [21], [32] in 2009 and 2010, respectively, ZIKV emerged in 2011 in the same area of the southeastern Senegal. The virus was detected from mosquitoes collected in all land cover classes sampled except indoor locations within villages, across a broad area, indicating widespread circulation. Moreover, ZIKV has also shown its ability to disperse further and invade human-inhabited areas from it southeastern Senegal focus. Indeed, it was detected in American scientists who became ill in the U.S. after working in Bandafassi, Senegal in 2008 [33].

ZIKV was detected in some mosquitoes in June, corresponding to the beginning of the rainy season in southern Senegal [21], [34]. This suggests rapid seasonal amplification, possibly due to efficient vertical transmission and/or maintenance in vertebrate reservoirs. Previous entomological surveillance at a forest gallery located 10 km from the city of Kédougou has shown that ZIKV can be isolated as early as July [20]. The early amplification of the virus could be facilitated by a short extrinsic incubation period, allowing relatively young mosquitoes to became transmission-competent. This observation is consistent with the results of an experimental study comparing the transmission of ZIKV and YFV by *Ae. aegypti*, which showed that 88% of the mosquitoes transmitted ZIKV but none transmitted YFV at day 7 post infection and only 36% of the mosquito transmitted YFV at day 14 post infection [15]. The amplification profile of ZIKV in Senegal presented as two phases, with an initial peak in June followed by another of greater amplitude between September and December. Given this amplification profile, surveillance of arboviruses in this area [35], [36] should be extended in time beyond standard sampling that focused only on July, October and November and may therefore have missed amplification events earlier in the season. Among arboviruses found in Southeastern Senegal, ZIKV has the highest annual frequency of amplifications detected in mosquitoes, including during 20 of the 34 years of surveillance between 1972 and 2005 [19].

Although monkeys living in forest canopy probably played a role in the ZIKV amplification, the rapid periodicity of amplification suggest that other vertebrates may also play an important role in ZIKV circulation. Consistent with this hypothesis, antibodies directed against ZIKV have been found in several vertebrate species [37]–[40], and its vectors have been found feeding on a diverse vertebrate fauna in small numbers of blood meals analyzed [41].

Assuming all infected mosquitoes were capable of transmission, ZIKV was transmitted by a large number of mosquito species; five of these vectors (*Ae. furcifer, Ae. taylori, Ae. luteocephalus, Ae. vittatus* and *Ae. africanus*) appear to play the most important roles in transmission. *Ae. furcifer, Ae. taylori, Ae. luteocephalus* were previously incriminated as the main vectors during sylvatic CHIK, DENV-2 and YF outbreaks in area [20], [21], [36], [42]. The lower involvement of *Ae. africanus* in previous ZIKV amplifications in the area may be due to the fact that collections were not previously conducted in the 2 forest sites where the species was abundantly found among 50 sites investigated [21]. The impact of ecological associations between vector species and individual arboviruses needs further clarification via laboratory studies. Some associations may be more efficient than the others in transmission in the enzootic cycle or may raise the risk of human exposure and epidemics.

ZIKV was isolated from *Ae. vittatus* in a Senegalese village for the first time in our study. We also report the first involvement of a mosquito species other than *Ae. furcifer* in arbovirus transmission in a domestic environment in southeastern Senegal. Previous failure to implicate *Ae. vittatus* in arbovirus transmission within domestic environments is discordant with the fact that this species readily feeds on humans and its larvae can be found within villages in southeastern Senegal and India [34], [43]. The detection of ZIKV in a pool of male *Ae. furcifer* suggests strongly that it is vertically transmitted in this species, and indicates that vertical transmission may be an important mechanism of local maintenance [19], [21], [42], [44]. Despite the fact that no infected *Ae. furcifer* females were collected within villages, the collection of the infected male nevertheless suggests that this species participates in the transmission of the ZIKV within villages.

The 2011 ZIKV amplification was widespread, involving all land cover classes investigated except indoor locations within villages. Transmission by several mosquito species was suggested to occur in different combinations, depending on the land cover class considered. This study suggests that very few villages were affected by this amplification and supports for the first time the involvement of *Ae. vittatus* as a bridge vector of ZIKV to humans within villages in southeastern Senegal. Our findings also suggest that vertical transmission of ZIKV by *Ae. furcifer* could be an important mechanism of ZIKV maintenance.

## Supporting Information

Table S1 UTM coordinates of sampling sites. ID: sampling sites identification.
(XLSX)

Table S2 Mosquitoes collected and tested for arboviruses infection, Kédougou, 2011.
(XLSX)

Table S3 Mosquito females infected by Zika virus, Kédougou, 2011.
(XLSX)
